# Risk factor analysis and nomogram development for advanced-stage hepatic fibrosis in patients with Wilson’s disease

**DOI:** 10.3389/fmed.2025.1650584

**Published:** 2025-07-30

**Authors:** Jiafeng Zhou, Zuolong Li, Junwei Wang, Zhenzhen Jiang, Tao Wang, Tianyu Xie, Liangchen Wang, Shuai Kang, Zhuang Tao, Meixia Wang

**Affiliations:** ^1^Encephalopathy Center, The First Affiliated Hospital of Anhui University of Chinese Medicine, Hefei, Anhui, China; ^2^Graduate School, Anhui University of Chinese Medicine, Hefei, Anhui, China; ^3^College of Resources and Environment, Anhui Agricultural University, Hefei, Anhui, China

**Keywords:** Wilson’s disease, predictive model, advanced hepatic fibrosis, risk factors, retrospective study

## Abstract

**Purpose:**

To investigate the risk factors for advanced-stage hepatic fibrosis in Wilson’s disease (WD), and developed a predictive nomogram to screen high risk patients with WD for early prevention and intervention.

**Methods:**

We retrospectively analyzed clinical data from WD in The First Affiliated Hospital of Anhui University of Chinese medicine between January 2010 and December 2024. Patients were divided into advanced hepatic fibrosis and non-advanced fibrosis groups according liver stiffness measurement. Identification of the independent risk factors for advanced hepatic fibrosis in WD was conducted through univariate and multivariate Cox regression analyses, followed by the construction of the clinical predictive model. The discriminative power, calibration, and clinical utility of the model were validated by receiver operating characteristic, calibration curves, and decision curve analysis (DCA).

**Results:**

The study cohort comprised 221 patients. Notably, CER, LN, HDL-C, TG, PLT, Sex, and Apo-A1 were identified as independent risk factors for advanced hepatic fibrosis in WD patients undergoing long-term maintenance therapy. The C-index demonstrated excellent discriminative capacity [training cohort: area under the curve (AUC) values of 0.918 at 36 months, 0.914 at 60 months, and 0.935 at 84 months; validation cohort: AUC values of 0.906, 0.917, and 0.888 at corresponding time points]. Calibration curves exhibited strong alignment between predicted and observed outcomes. The DCA quantified clinical benefit probability thresholds across varying time intervals.

**Conclusion:**

The nomogram predictive model demonstrated high accuracy and provides a practical tool for the early identification and risk prediction of advanced hepatic fibrosis in WD patients undergoing long-term maintenance therapy.

## 1 Introduction

Wilson’s disease (WD) is a rare, autosomal recessive genetic disorder resulting from mutations in the ATP7B gene. These mutations disrupt normal hepatic copper metabolism, leading to the accumulation of copper in the liver and subsequent deposition in extrahepatic tissues. The clinical presentation typically included hepatic manifestations—ranging from asymptomatic hepatomegaly to acute or chronic liver failure—progressive extrapyramidal symptoms, neuropsychiatric disturbances, and the presence of Kayser–Fleischer rings due to corneal copper deposition. Additional common clinical presentations include hemolytic anemia, osteoarticular manifestations, and hematuria (1). The liver, which is the primary organ involved in copper metabolism, is pathologically impaired during the early stages of WD, resulting in copper accumulation. Accumulation of copper subsequently initiates hepatocytic inflammatory responses, including autophagy and apoptosis (2, 3). Pathologically, WD is a progressive disorder: notably, at the initial stages, it is predominantly characterized by hepatocytic lipid droplet accumulation and steatosis, leading to hepatic inflammatory responses and fibrosis, and culminating in liver cirrhosis (4). Notably, clinical studies indicate that approximately 35%–45% of WD patients—including asymptomatic patients and those with various clinical phenotypes—present with established cirrhosis at the time of diagnosis (3, 5), with cirrhosis-related complications emerging as primary contributors to disease-related mortality (4).

Hepatic fibrosis, characterized by the pathological deposition of extracellular matrix components in response to chronic liver injury, represents both a pivotal intermediate stage in the progression of WD-related hepatic pathology toward cirrhosis and a potentially reversible pathological phase within the spectrum of liver damage (6, 7). Clinical evidence has shown that timely intervention can effectively inhibit or reverse fibrotic progression. However, this requires stage-specific therapeutic strategies, including anti-inflammatory and hepatoprotective therapies during the initial stages, and intensified targeted antifibrotic regimens during advanced phases (8). Notably, clinical practice guidelines established by the European Association for the Study of the Liver (EASL) (9) indicate that patients with advanced hepatic fibrosis exhibit significantly elevated risks of decompensated cirrhosis and hepatocellular carcinoma (HCC), thereby underscoring the need for timely intervention to improve the prognostic outcomes of the disease.

Advanced clinical predictive models employing machine learning technology have significantly enhanced the accuracy of dynamic risk assessment by systematically integrating multidimensional clinical patient data (10). Notably, compared to traditional univariate assessment approaches, this model effectively captures complex interactions among multifactorial parameters, demonstrating superior sensitivity and specificity in predicting the progression of hepatic fibrosis. Despite the initial progress in the development of predictive models for WD-related hepatic conditions—such as the hepatic fibrosis prediction model for patients with lipid metabolism disorders established by Zhao et al. (11) and the serum non-ceruloplasmin-bound copper prediction model developed by Tao et al. (12)—a significant gap remains in the availability of robust, quantitative predictive tools for advanced hepatic fibrosis. Consequently, this study aims to develop a risk prediction model for advanced hepatic fibrosis through retrospective cohort analysis, incorporating baseline clinical parameters and medical history characteristics of WD patients. This model is anticipated to provide quantitative evidence for the early identification of high-risk patients, thereby informing the development of personalized intervention strategies and ultimately improving the long-term prognosis for patients with WD. Notably, the predictive model is developed by strategically incorporating routine laboratory indices to ensure clinical applicability, which aligns with practical healthcare requirements.

## 2 Materials and methods

### 2.1 Enrollment of participants

This was a retrospective study involving the collection of routine admission laboratory parameters and medical history data of WD patients receiving long-term maintenance therapy at the First Affiliated Hospital of Anhui University of Chinese Medicine between January 2010 and December 2024. The sample size was 221 patients, determined using the formula


$$n=z^{2}\,s^{2}/d^{2}$$

where *z* = confidence interval, *n* = sample size, *d* = margin of error, and σ = standard deviation set to 0.5. However, this sample size was also dependent on the maximum patient capacity of the medical center.

Patients were randomly assigned to either a training (70%) or a validation (30%) cohort using the random number table method. Based on the guidelines “Guidelines for the prevention and treatment of metabolic dysfunction-associated (non-alcoholic) fatty liver disease (version 2024)” (13), patients were stratified into a non-advanced hepatic fibrosis group [liver stiffness measurement (LSM) ≤ 12.0 kPa] and an advanced hepatic fibrosis group (LSM > 12.0 kPa). The protocols employed in this study were approved by the Medical Ethics Committee of the First Affiliated Hospital of Anhui University of Chinese Medicine (Approval No. 2024AH-49), with strict adherence to all ethical standards.

### 2.2 Inclusion criteria

Patients diagnosed with WD based on the “Guidelines for the diagnosis and treatment of hepatolenticular degeneration (2022 edition)” (14), with naïve treatment, non-advanced hepatic fibrosis, age-unrestricted, and complete medical records; and patients undergoing long-term standardized copper-chelating therapy following confirmed diagnosis.

### 2.3 Exclusion criteria

Patient newly diagnosed with WD; presence of fulminant liver failure (with or without hemolytic anemia) and decompensated cirrhosis; patients exhibiting cognitive dysfunction (Mini-Mental State Scale score of ≤22 or Montreal Cognitive Assessment Scale score of <26); patients with severe neurological impairment, such as torsion spasms; patients with concurrent mental illness; patients exhibiting moderate to severe or complete dependence in performing daily life activities (Barthel index rating scale ≤ 70 points); patients presenting with serious diseases (opportunistic infections, tumors, and blood system diseases); pregnant or lactating patients; patients with incomplete medical records.

### 2.4 Clinical data collection and testing methods

We collected baseline data of treatment-naïve patients and their most recent hospitalization data in the traceable initial encounter, and the details are as follows: general data collected included gender, age, marital status, allergies, disease course, clinical classification, history of splenectomy, and treatment duration. Laboratory analyses were conducted on the following: white blood cell (WBC), red blood cell (RBC), hemoglobin (HGB), platelet (PLT), reticulocyte (Ret), alanine aminotransferase (ALT), aspartate aminotransferase (AST), gamma-glutamyl transferase (GGT), total bilirubin (TBIL), direct bilirubin (DBIL), indirect bilirubin (IBIL), blood urea nitrogen (BUN), serum creatinine (Scr), total cholesterol (TC), triglycerides (TG), high-density lipoprotein cholesterol (HDL-C), low-density lipoprotein cholesterol (LDL-C), apolipoprotein A1 (Apo-A1), apolipoprotein B (Apo-B), lipoprotein(a) (Lpa), cystatin C (CysC), type IV collagen (CIV), hyaluronic acid (HA), laminin (LN), procollagen III N-terminal peptide (PIIINP), urinary immunoglobulin G (IgGU), urinary transferrin (TRU), urinary microalbumin (MA), urinary α1-microglobulin (α1MG), urinary β2-microglobulin (β2MG), 24-h urinary copper excretion, 24-h urinary zinc excretion, 24-h urinary calcium excretion, 24-h urinary magnesium excretion, serum copper, serum zinc, serum calcium, serum magnesium, ceruloplasmin (CER), and non-ceruloplasmin-bound copper (NCBC). The LSM level from the FibroScan were recorded in their most recent hospitalization data.

Blood samples were collected from fasting patients via morning venipuncture on the day following hospital admission using sodium citrate vacuum anticoagulant tubes. Subsequent biochemical analyses were performed by certified laboratory technicians at the hospital’s central laboratory using standardized automated analyzers. For 24-h urinary quantification of copper, zinc, calcium, and magnesium, patients were instructed to void and discard the initial urine sample at 7:00 a.m., followed by continuous collection of all subsequent urine over the next 24 h into prerinsed containers, which were evenly divided into three aliquots; total urine volume was recorded after the collection period (7:00 a.m. the following day), and 5 ml aliquots were extracted and submitted for biochemical analysis. The urinary five-protein panel (IgGU, TRU, MA, α1MG, and β2MG) necessitated a 24-h urine collection with precise volume measurement. Samples of 8 ml were preserved at −40°C and subsequently analyzed by designated personnel. A Hitachi ARIETTA 850 ultrasound system equipped with a C252 convex array probe (3–6 MHz) in abdominal mode was used to conduct LSM. After fasting for ≥8 h, patients were positioned supine with quiet breathing. Experienced sonographers (with ≥50 valid elastography examinations within the past 6 months) performed measurements in the right hepatic lobe (preferably segment S5), 1–2 cm beneath the Glisson’s capsule, ensuring avoidance of vascular structures. During the measurement process, patients were instructed to hold their breath for 3–4 s after normal expiration, with the probe maintained perpendicular to the liver surface. The system software automatically calculated shear wave speed (SWM, m/s) and LSM (kPa). Quality control required valid vibration metrics (valid scan number and valid number ≥50%). Five valid measurements were obtained per patient, with the median value adopted as the final result.

### 2.5 Statistical analysis

Statistical analyses were performed using SPSS (version 26.0) and R programming language (version 4.0.3). Continuous variables were expressed as median (interquartile range, IQR), with analysis conducted using the Mann–Whitney *U* test. Categorical variables were presented as frequency (%), with analysis performed using the Chi-square test. A *P*-value of <0.05 was considered to be statistically significant. Univariate Least Absolute Shrinkage and Selection Operator (LASSO) regression and Cox proportional hazards models were used to reduce the dimensionality of the clinical feature dimensions and identify predictors with non-zero coefficients associated with advanced hepatic fibrosis in patients with WD undergoing maintenance therapy. Multivariate Cox regression was used to further screen the independent risk factors, which was then followed by the construction of a clinical prediction model for individualized risk estimation. The performance of these models was evaluated using the receiver operating characteristic (ROC) curve analysis (area under the curve, AUC), calibration curves, and decision curve analysis (DCA). These evaluation techniques collectively assessed discriminative power, calibration accuracy, and clinical utility in both internal and external validation.

## 3 Results

### 3.1 Patient characteristics

A total of 221 WD patients receiving long-term maintenance therapy were enrolled in this study, with 155 cases in the training cohort and 66 cases in the validation cohort. Notably, no significant differences were observed in baseline characteristics for both groups involving clinical parameters such as demographic data, laboratory profiles, and imaging metrics (*P* > 0.05). Detailed patient characteristics are presented in Table 1.

**TABLE 1:** Clinical characteristics comparison between training and validation cohorts.

Characteristics	Validation cohort *N* = 66	Training cohort *N* = 155	*P*	Characteris-tics	Validation cohort *N* = 66	Training cohort *N* = 155	*P*
Sex			1.000	HDL-C (mmol/L)	1.21 [1.02, 1.46]	1.24 [1.08, 1.51]	0.637
Male	42 (63.6%)	100 (64.5%)		LDL-C (mmol/L)	2.23 [1.86, 2.66]	2.20 [1.81, 2.78]	0.850
Female	24 (36.4%)	55 (35.5%)		Apo-A1 (g/L)	1.23 [1.05, 1.42]	1.32 [1.16, 1.45]	0.063
Age (year)	21.0 [16.0, 31.5]	25.0 [17.0, 33.0]	0.415	Apo-B (g/L)	0.71 [0.59, 0.87]	0.70 [0.55, 0.86]	0.380
Disease course (months)	96.0 [71.2, 132]	108 [72.0, 168]	0.265	Lpa (mg/L)	63.3 [25.2, 130]	42.8 [19.8, 98.3]	0.099
Clinical classification			0.845	CysC (mg/L)	0.95 [0.84, 1.10]	0.94 [0.83, 1.10]	0.922
Hepatic injury	31 (47.0%)	67 (43.2%)		CIV (ng/ml)	55.6 [34.1, 90.8]	54.2 [33.4, 87.9]	0.993
Neurological symptomatology	25 (37.9%)	65 (41.9%)		HA (ng/ml)	72.6 [48.2, 186]	85.4 [50.6, 147]	0.945
Mixed hepatic-neurologic type	10 (15.2%)	23 (14.8%)		LN (ng/ml)	96.0 [40.1, 164]	84.5 [39.0, 137]	0.259
Marital status			0.898	PIIINP (ng/ml)	17.4 [11.9, 23.2]	13.8 [9.39, 22.1]	0.064
Unmarried/divorce	46 (69.7%)	105 (67.7%)		IgGU (mg/L)	5.66 [3.88, 10.1]	5.66 [3.65, 8.54]	0.489
Married/cohabitation	20 (30.3%)	50 (32.3%)		TRU (mg/L)	2.12 [0.54, 2.24]	2.13 [1.06, 2.24]	0.122
Allergies			0.542	MA (mg/L)	11.0 [8.57, 12.5]	11.0 [10.3, 12.2]	0.482
Yes	9 (13.6%)	28 (18.1%)		α1MG (mg/L)	11.1 [5.58, 21.8]	11.1 [5.78, 18.5]	0.790
No	57 (86.4%)	127 (81.9%)		β2MG (mg/L)	0.52 [0.20, 1.03]	0.59 [0.20, 1.82]	0.691
Splenectomy			0.529	24-h urinary copper excretion (μg/24 h)	900 [501, 1363]	863 [616, 1211]	0.788
Yes	9 (13.6%)	15 (9.68%)		24-h urinary zinc excretion (μg/24 h)	3,860 [2,061, 5,967]	3,304 [1,808, 5,028]	0.383
No	57 (86.4%)	140 (90.3%)		24-h urinary calcium excretion (μg/24 h)	179 [110, 254]	177 [104, 270]	0.907
Treatment duration (months)	62.5 [35.0, 86.8]	59.0 [36.0, 92.0]	0.653	24-h urinary magnesium excretion (μg/24 h)	71.7 [52.8, 101]	72.0 [52.5, 90.0]	0.453
WBC (×10^9^/L)	5.16 [3.68, 6.98]	4.70 [3.56, 5.82]	0.198	Serum copper (μmol/L)	2.81 [1.90, 5.90]	3.45 [1.84, 5.90]	0.408
RBC (×10^12^/L)	4.47 [4.21, 4.75]	4.44 [4.19, 4.83]	0.692	serum zinc (μmol/L)	19.3 [14.3, 21.6]	17.7 [13.2, 21.6]	0.317
HGB (g/L)	126 [121, 135]	127 [119, 136]	0.984	Serum calcium (μmol/L)	2.12 [2.12, 2.18]	2.12 [2.10, 2.15]	0.055
PLT (×10^9^/L)	180 [113, 281]	162 [102, 250]	0.392	Serum magnesium (μmol/L)	0.82 [0.77, 0.82]	0.82 [0.76, 0.82]	0.544
Ret (×10^12^/L)	0.05 [0.05, 0.07]	0.05 [0.04, 0.07]	0.758	CER (g/L)	0.07 [0.03, 0.08]	0.07 [0.03, 0.08]	0.766
ALT (U/L)	30.5 [20.2, 63.5]	28.0 [18.0, 55.0]	0.164	NCBC (mg/L)	0.06 [-0.10, 0.12]	0.05 [−0.07, 0.13]	0.614
AST (U/L)	31.0 [22.2, 40.3]	27.0 [20.0, 36.1]	0.084	Sheth	0.88 [0.61, 1.19]	0.93 [0.67, 1.25]	0.255
GGT (U/L)	34.0 [23.0, 49.8]	30.0 [18.5, 50.0]	0.336	FIB-4	0.67 [0.34, 1.38]	0.84 [0.39, 1.43]	0.358
TBIL (μmol/L)	13.0 [8.76, 17.6]	11.6 [8.10, 16.4]	0.381	APRI	0.42 [0.31, 0.70]	0.44 [0.28, 0.72]	0.599
DBIL (μmol/L)	3.46 [2.50, 5.39]	3.30 [2.20, 5.68]	0.584	GPR	0.32 [0.20, 0.52]	0.30 [0.18, 0.64]	0.750
IBIL (μmol/L)	9.38 [5.42, 12.2]	8.20 [5.79, 11.2]	0.370	Copper to zinc ratio in 24-h urinary excretion	0.22 [0.12, 0.47]	0.27 [0.13, 0.52]	0.346
BUN (mmol/L)	4.27 [3.55, 5.29]	4.59 [3.71, 5.60]	0.236	Copper to zinc ratio in serum	0.16 [0.10, 0.27]	0.26 [0.11, 0.27]	0.051
Scr (μmol/L)	50.2 [37.9, 67.9]	54.2 [40.0, 71.9]	0.254	Fibrosis			0.799
TC (mmol/L)	3.84 [3.50, 4.79]	3.99 [3.40, 4.67]	0.684	Advanced hepatic fibrosis	14 (21.2%)	37 (23.9%)	
TG (mmol/L)	0.98 [0.73, 1.45]	1.01 [0.72, 1.38]	0.995	Non-advanced hepatic fibrosis	52 (78.8%)	118 (76.1%)	

WBC, white blood cell; RBC, red blood cell; HGB, hemoglobin; PLT, platelet; Ret, reticulocyte; ALT, alanine aminotransferase; AST, aspartate aminotransferase; GGT, gamma-glutamyl transferase; TBIL, total bilirubin; DBIL, direct bilirubin; IBIL, indirect bilirubin; BUN, blood urea nitrogen; Scr, serum creatinine; TC, total cholesterol; TG, triglycerides; HDL-C, high-density lipoprotein cholesterol; LDL-C, low-density lipoprotein cholesterol; Apo-A1, apolipoprotein A1; Apo-B, apolipoprotein B; Lpa, lipoprotein(a); CysC, cystatin C; CIV, type IV collagen; HA, hyaluronic acid; LN, laminin; PIIINP, procollagen III N-terminal peptide; IgGU, urinary immunoglobulin G; TRU, urinary transferrin; MA, urinary microalbumin; α1MG, urinary α1-microglobulin; β2MG, urinary β2-microglobulin; CER, ceruloplasmin; FIB-4, fibrosis 4 score; APRI, aspartate aminotransferase to platelet ratio index; GPR, gamma-glutamyl transpeptidase to platelet ratio.

### 3.2 Univariate regression analysis

**FIGURE 1:**
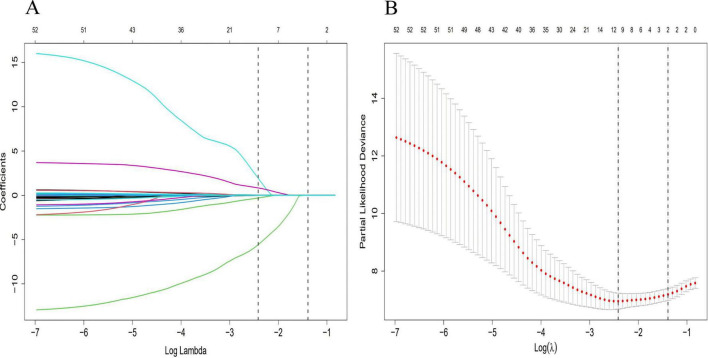
Diagram of univariate LASSO regression analysis. **(A)** Log_lambda and path coefficient diagram from LASSO regression analysis. **(B)** Diagram of CV_LASSO. CER, 24-h urinary calcium excretion, LN, HDL-C, TG, PLT, Sex, Ret, IBIL, Apo-A1, and α1MG were related factors for advanced hepatic fibrosis.

Univariate LASSO regression and Cox proportional hazards analyses were performed on clinical characteristics for the training cohort to identify factors associated with the development of advanced hepatic fibrosis for WD patients undergoing long-term maintenance therapy. Following the elimination of redundant dimensions, the significant predictors identified included CER, 24-h urinary calcium excretion, LN, HDL-C, TG, PLT, Sex, Ret, IBIL, Apo-A1, and α1MG (*P* < 0.1). The variable selection process using the LASSO regression analysis is detailed in Figure 1.

### 3.3 Multivariate regression analysis

**FIGURE 2:**
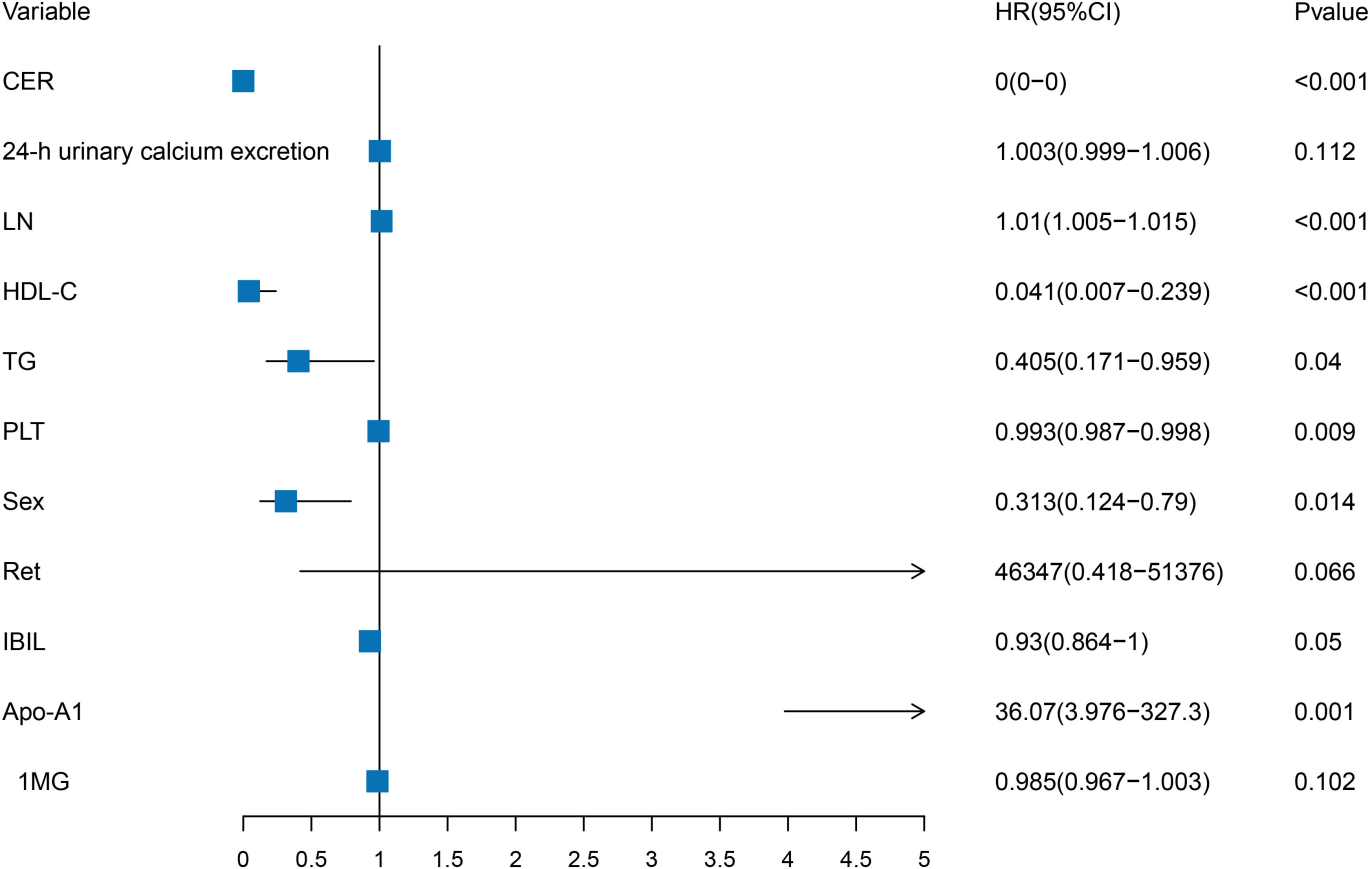
Forest plot of independent risk factors in multivariate Cox proportional hazards analysis. CER, LN, HDL-C, TG, PLT, Sex, and Apo-A1 were independent risk factors for advanced hepatic fibrosis.

Clinical variables identified using the univariate LASSO regression and Cox proportional hazards analyses were incorporated into the multivariate Cox analyses. The results indicated that CER, LN, HDL-C, TG, PLT, Sex, and Apo-A1 were independent risk factors for advanced hepatic fibrosis in WD patients undergoing long-term maintenance therapy (*P* < 0.05). Detailed results of the multivariate Cox proportional hazard analyses are presented in Table 2 and Figure 2.

**TABLE 2:** Multivariate Cox proportional hazards analysis of independent risk factors.

Characteristics	*B*	SE	HR	CI	*Z*	*P*
CER (g/L)	−38.194	7.723	<0.001	<0.001 to <0.001	−4.946	<0.001
24-h urinary calcium excretion (μg/24 h)	0.003	0.002	1.003	0.999–1.006	1.588	0.112
LN (μg/24 h)	0.01	0.002	1.010	1.005–1.015	4.206	<0.001
HDL-C (mmol/L)	−3.204	0.904	0.041	0.007–0.239	−3.544	<0.001
TG (mmol/L)	−0.904	0.44	0.405	0.171–0.959	−2.056	0.040
PLT (×10^9^/L)	−0.007	0.003	0.993	0.987–0.998	−2.621	0.009
Sex	−1.163	0.473	0.313	0.124–0.79	−2.459	0.014
Ret (×10^12^/L)	13.046	7.101	463,470.92	0.418–513,767,381,830.178	1.837	0.066
IBIL (μmol/L)	−0.073	0.037	0.930	0.864–1	−1.956	0.050
Apo-A1 (g/L)	3.586	1.125	36.079	3.976–327.388	3.187	0.001
α1MG (mg/L)	−0.015	0.009	0.985	0.967–1.003	−1.633	0.102

CER, ceruloplasmin; LN, laminin; HDL-C, high-density lipoprotein cholesterol; TG, triglycerides; PLT, platelet; Ret, reticulocyte; IBIL, indirect bilirubin; Apo-A1, apolipoprotein A1; α1MG, urinary α1-microglobulin.

### 3.4 Establishment and visualization of the nomogram model

**FIGURE 3:**
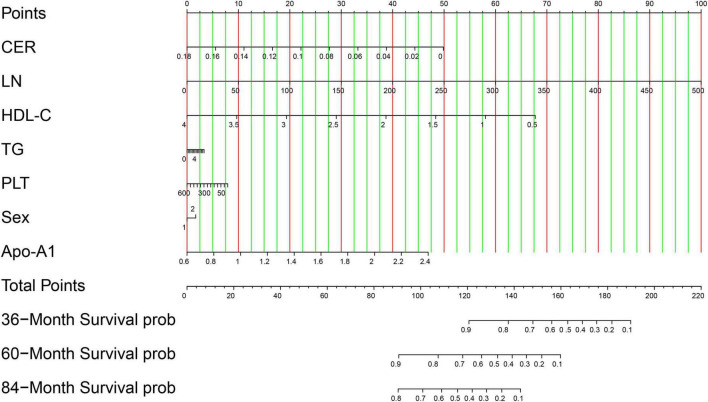
Visualization of the nomogram model. Variables are displayed on the left side, whereas scores are on the right. The total score is calculated by summing the values of each variable. The probability of the occurrence of advanced hepatic fibrosis can be predicted using the linear predictor line. In validation cohort, patient (ID: 46) with CER 0.033 g/L, LN 143.54 ng/ml, HDL-C 1.51 mmol/L, TG 0.86 mmol/L, PLT 88 × 10^9^/L, Sex man, and Apo-A1 0.9 g/L, the scores of each variable is 41.25, 28.75, 48.75, 0.8, 6.25, 0, and 8 based on the top points line, with total scores is 133.8. The probability of the occurrence of advanced hepatic fibrosis is 85% in 36-month treatment intervals using the linear predictor line and the total points line.

The seven identified independent risk factors were successfully incorporated to develop a nomogram predictive model for the development of advanced hepatic fibrosis in WD patients receiving long-term maintenance therapy. A visual representation of the nomogram is presented in Figure 3. Using the scoring scale located along the top axis of the model, clinicians can assign individual scores corresponding to each of the seven risk factors. The sum of these scores yields a total point value, which corresponds to the predicted probability of advanced hepatic fibrosis, as indicated on the model’s bottom probability axis. This allows for an intuitive, point-based estimation of fibrosis risk in individual patients.

### 3.5 Nomogram performance evaluation and validation

#### 3.5.1 Concordance index

**FIGURE 4:**
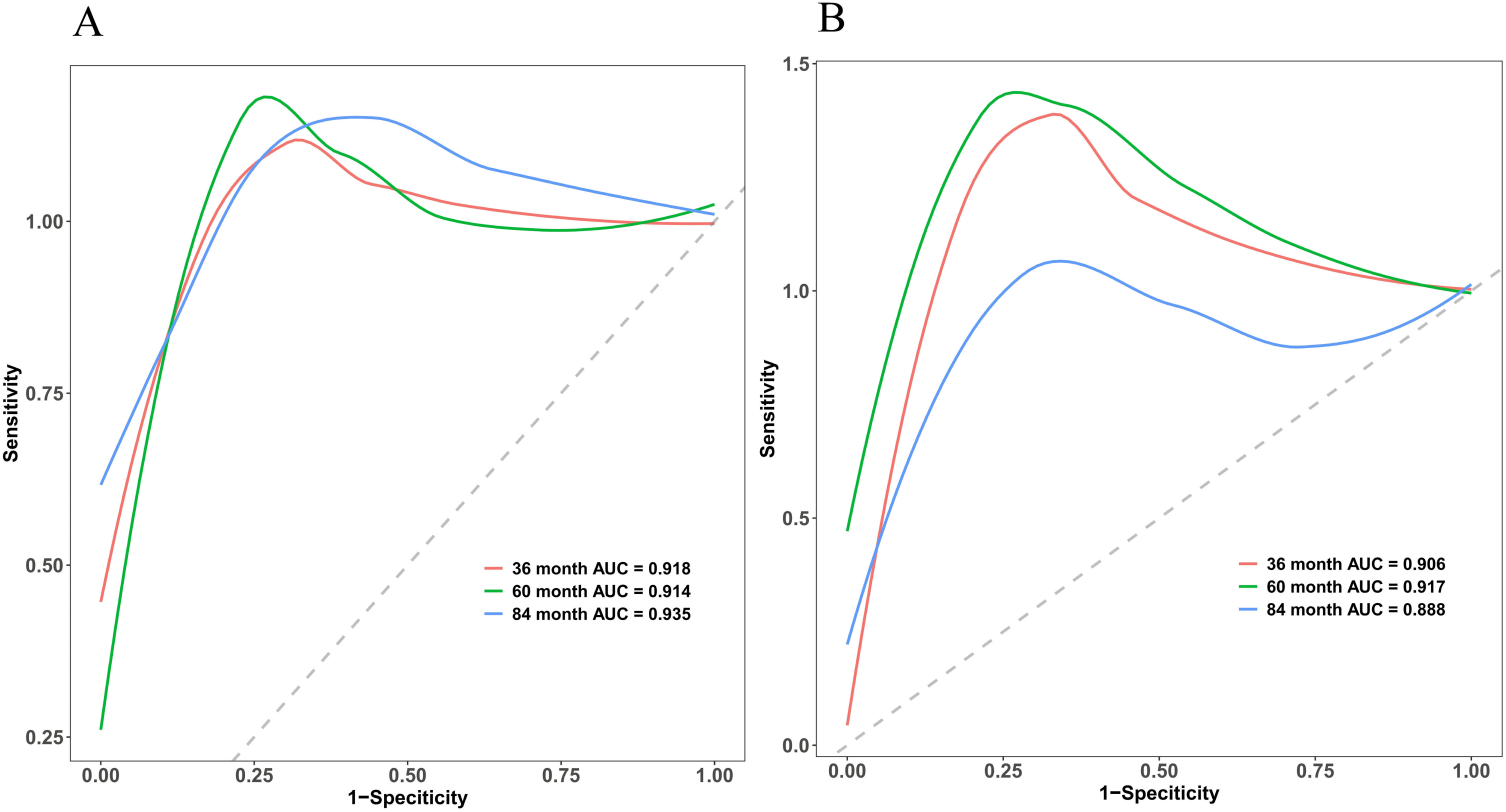
Multi-temporal ROC analysis of the nomogram model. The *x*-axis representing 1-specificity and the *y*-axis sensitivity. The red, green, and blue curves correspond to 36-month, 60-month, and 84-month treatment intervals, respectively. The AUC values demonstrated robust discriminative performance: 0.918 (36 months), 0.914 (60 months), and 0.935 (84 months) in the training cohort **(A)**, and 0.906, 0.917, and 0.888 in the validation cohort **(B)**, respectively. The model’s high discriminative capacity was further confirmed by its C-index.

The discriminative power of the model was assessed using the concordance index (C-index). Specifically, this metric differentiates between outcomes. Notably, a C-index of 0.50–0.70 indicates low discriminative power; 0.71–0.90 indicates moderate discriminative power; and values >0.90 demonstrate high discriminative power. The nomogram developed in this study demonstrated superior discriminative performance, with an AUC of 0.918 at 36 months, 0.914 at 60 months, and 0.935 at 84 months in the training cohort, while the validation cohort exhibited an AUC of 0.906, 0.917, and 0.888, respectively. Considering potential overfitting and optimism bias, we further performed a bootstrap validation (500 repetitions) on the entire dataset, showing that the C-index remains stable between 0.80 and 0.90 with 95% confidence intervals (0.812–0.902). Detailed C-index measurements are presented in Figure 4 and Supplementary Figure 1.

#### 3.5.2 Calibration curves

**FIGURE 5:**
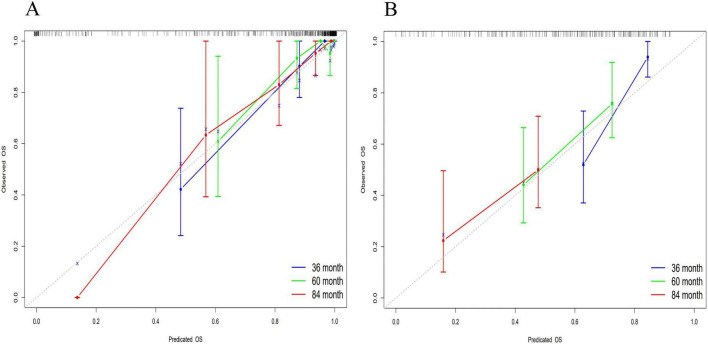
Multi-temporal calibration curves of the nomogram model. The *x*-axis representing predicted probabilities and the *y*-axis indicating observed frequencies of advanced hepatic fibrosis. The black dashed line represents ideal prediction accuracy, while blue, green, and red curves correspond to 36-month, 60-month, and 84-month treatment intervals, respectively. Excellent model performance is indicated by close alignment of these curves to the reference line. In both training **(A)** and validation **(B)** cohorts, the calibration curves exhibited strong concordance with the ideal prediction line, demonstrating excellent consistency and calibration accuracy across all timepoints.

The accuracy of the nomogram model was evaluated using the calibration curve. Notably, in this study, the calibration curve was closely aligned with the reference line, indicating excellent consistency and calibration. Details of calibration analyses are presented in Figure 5.

#### 3.5.3 Decision curve analysis

**FIGURE 6:**
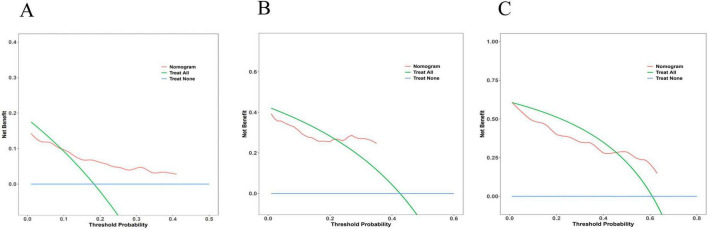
Decision curve analysis for the nomogram model. The *x*-axis representing threshold probabilities and the *y*-axis indicating net benefit. The blue line reflects net benefit when all patients develop advanced hepatic fibrosis, the green line represents net benefit assuming no fibrosis progression, and the red line denotes model-derived net benefit. Clinically beneficial threshold probability ranges were identified as 0.093–0.410 at 36 months **(A)**, 0.217–0.349 at 60 months **(B)**, and 0.455–0.630 at 84 months **(C)**, where the red line consistently surpassed the “treat-all” and “treat-none” strategies.

The DCA was employed to evaluate the clinical utility of the nomogram model by quantifying the net benefit analysis across various probability thresholds. The model demonstrated clinically actionable prediction ranges for advanced hepatic fibrosis risk: 0.093–0.410 at 36 months, 0.217–0.349 at 60 months, and 0.455–0.630 at 84 months, indicating significant clinical benefit within these probability intervals. Detailed DCA trajectories are presented in Figure 6.

#### 3.5.4 Kaplan–Meier analysis for risk stratification

**FIGURE 7:**
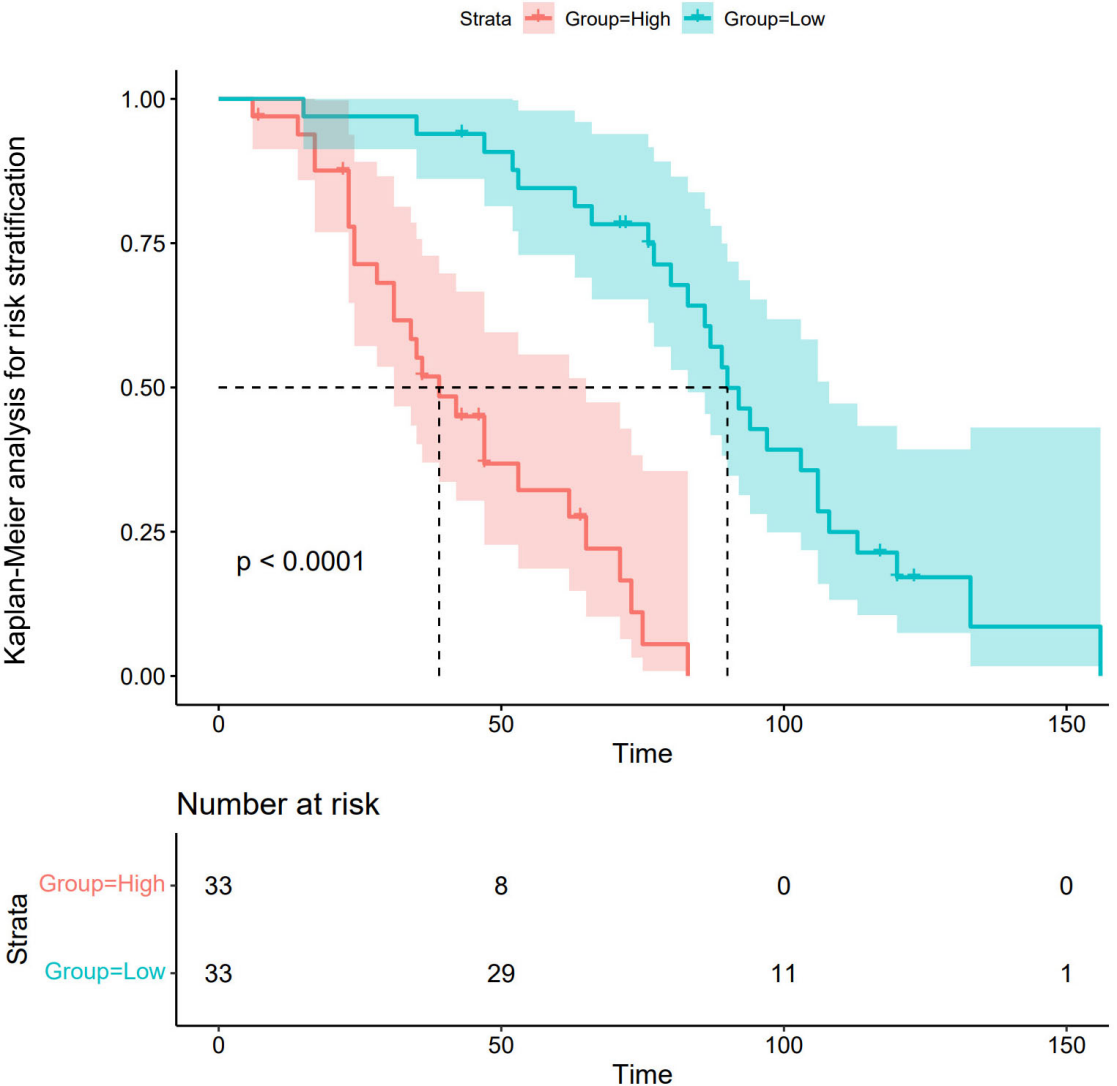
Kaplan–Meier analysis for risk stratification. Green lines and shaded areas represent the probability of disease progression with 95% confidence intervals (CIs) for the low-risk group. The red lines and shaded areas indicate the corresponding values for the high-risk group. The high-risk group demonstrated a significantly higher likelihood of disease progression compared to the low-risk group.

Risk stratification of the nomogram prediction model stratified participants into high- and low-risk groups. Notably, statistically significant differences in disease progression-free probability were observed between the cohorts (log-rank *P* < 0.001). The results revealed a statistically significant disparity between the two groups, with the high-risk group demonstrating a significantly higher likelihood of disease progression compared to the low-risk group. Detailed methodological and outcome specifications of this validation assessment are illustrated in Figure 7.

## 4 Discussion

In this study, we identified CER, Apo-A1, LN, PLT, HDL-C, TG, and Sex as independent risk factors. Subsequently, these factors were employed to construct a predictive model for assessing the risk of advanced hepatic fibrosis in WD patients undergoing long-term maintenance therapy.

Copper is a trace element involved in various biological processes, such as the regulation of lipid metabolism, which helps in maintaining cellular homeostasis. Research has indicated that pathological accumulation of copper in the liver in WD patients inhibits nuclear receptor function, leading to lipid metabolism disorders and dysfunction of mitochondrial energy synthesis, thereby inducing hepatocyte injury (15). Notably, imbalances in free fatty acids caused by lipid metabolism abnormalities exacerbate pathological progression of excessive free fatty acids, which directly contribute to abnormal lipid droplet deposition in hepatocytes through lipotoxic effects, thereby promoting hepatic steatosis (16, 17). The 2020 Asia-Pacific guidelines for Metabolically Associated Fatty Liver Disease (MAFLD) explicitly classified WD-related liver disease as secondary fatty liver disease (18), indicating that WD exhibited comparable molecular mechanisms such as lipid peroxidation and endoplasmic reticulum stress. Given the current absence of established non-invasive diagnostic thresholds specifically tailored to advanced hepatic fibrosis in WD, reduction of the effective “event” based on combined threshold, and LSM has been validated as a mature and relatively stable indicator, this study established a single LSM threshold for advanced hepatic fibrosis applicable to WD patients (13).

This study identified CER, a diagnostic criterion for WD, as an independent risk factor for advanced hepatic fibrosis progression in WD. The biosynthesis of CER—a hepatogenic α2-glycoprotein—is regulated by inflammatory cytokine-mediated transcriptional control. Notably, serum CER levels transiently increase through stress compensation mechanisms during acute inflammation or tissue injury (19). Conversely, these levels exhibit progressive decline with deteriorating hepatic synthetic function as fibrosis advances to cirrhosis (from compensated to decompensated stages) (19, 20). This bidirectional dynamic fluctuation, closely associated with hepatic reserve capacity, positions a CER as a pivotal biomarker for evaluating transitional phases in the progression of hepatic fibrosis.

Notably, the metabolic characteristics of Apo-A1, another key predictor, provide complementary assessment dimensions to CER. This lipoprotein is synthesized through the hepatic-intestinal axis, and research first documented its association with chronic liver disease in 1986 (21). Clinically, Apo-A1 is routinely combined with alpha-2-macroglobulin (A2M) and haptoglobin (HP) to facilitate monitoring of the progression of the liver disease (22). During the decompensated phase of cirrhosis, pathological alterations including hepatocyte swelling, necrosis, and steatosis reduce Apo-A1 synthesis efficiency by >70%. Furthermore, the expression of this lipoprotein is significantly reduced before clinical manifestations of hepatic decompensation. Mechanistically, this phenomenon is linked to the “molecular trap” effect caused by collagen deposition in hepatic sinusoidal endothelial cells during advanced fibrosis stages, resulting in increased entrapment or consumption of Apo-A1 (23). This sensitive, preclinical decline—occurring before conventional liver function abnormalities—underscores apolipoprotein A1 (Apo-A1)’s distinct utility as an early warning biomarker for hepatic fibrosis and its potential role in disease stratification.

The dynamic monitoring of these two biomarkers demonstrates significant clinical synergy: CER indicates the homeostatic equilibrium of hepatic compensatory reserve through its bidirectional fluctuations, while Apo-A1 reveals early structural alterations in the hepatic sinusoid microenvironment via its preemptive signaling properties. This multidimensional laboratory evidence framework enables precise quantification of hepatocyte injury severity, as well as offering an integrated biomarker approach, evaluation of therapeutic efficacy, and prognostic assessment in WD-associated hepatic fibrosis.

Extracellular matrix contains LN, which is an important non-collagenous structural glycoprotein. It is primarily synthesized by hepatic stellate cells (HSCs), endothelial cells, and biliary epithelial cells. Notably, LN comprises the core components of the basement membrane and mediates cellular adhesion, migration, and signal transduction (24, 25). Under physiological conditions, the expression level of LN in liver tissue is relatively low (24). However, upon damage to liver cells, activation of HSC abnormally promotes the thickening of the basement membrane and the formation of fibrous septa through LN secretion. This process can trigger the “capillarization” of hepatic sinusoids and portal hypertension. Concurrently, it can lead to a significant increase in the serum LN levels (24, 26). Notably, upregulation of LN is pathologically harmful and contributes to the progression of hepatic fibrosis, as it contributes to the remodeling of the extracellular matrix. The upregulation of biomarkers such as serum LN and HA is a direct indicator of the imbalance in extracellular matrix metabolism (26–28). This study not only confirms that increase in the serum LN level is related to the progression of hepatic fibrosis in patients with WD, but also demonstrate the dynamic development characteristics of hepatic fibrosis from “point to surface,” further highlighting the potential value of LN as a noninvasive biomarker in the early identification of hepatic fibrosis in the advanced stage of WD and the monitoring of progression of this condition.

Imbalance of PLT in WD-associated hepatic fibrosis manifests dual pathological effects. On one hand, disruption of hepatic sinusoid architecture—such as capillarization mediated by LN-induces hypersplenism and reduced thrombopoietin (TPO) synthesis, resulting in the reduction of PLT (29, 30). Conversely, copper accumulation-related oxidative stress and inflammatory cytokine release (such as TNF-α and IL-1β) impair PLT functionality and suppress their antifibrotic roles, including hepatic regeneration mediated by HGF/VEGF and inhibition of HSC activation (31–34). Critically, PLT depletion and dysfunction exacerbate LN-mediated extracellular matrix deposition, contributing to fibrogenesis. Portal hypertension intensifies hypersplenism, perpetuating a pathological feedback loop, while reduced secretion of PLT-derived reparative factors further impairs hepatic regenerative capacity (35), accelerating the transition to irreversible fibrosis. This LN–PLT interaction network not only provides a dual-dimensional biomarker system (“structural remodeling-functional compensation”) for predicting advanced hepatic fibrosis in WD but also establishes a theoretical foundation for combinatorial targeting of extracellular metabolism and modulation of PLT function (such as TPO agonists or antifibrotic factor delivery).

In this study, HDL-C and TG were identified and established as indispensable factors for the construction of the predictive model. Specifically, these factors drive insulin resistance and inflammatory cascades through “copper-lipid co-toxicity,” which constitutes the core metabolic axis for fibrosis progression. In WD patients, the liver—a key insulin-responsive organ—develops mitochondrial dysfunction and oxidative stress due to copper homeostasis imbalance, thereby inducing systemic glucose-lipid metabolic dysregulation (36). Specifically, hepatic insulin resistance attenuates the inhibitory effect of insulin on gluconeogenesis, while copper accumulation abnormally activates lipid synthesis pathways such as sterol regulatory element-binding protein 1c (SREBP-1c), leading to hepatocyte lipid deposition (15, 37). This copper-lipid co-toxic milieu directly damages hepatocytes and accelerates fibrosis through multiple mechanisms: (1) mitochondrial reactive oxygen species (ROS) overproduction: copper overload induces excessive mitochondrial ROS, activating the NF-κB signaling pathway, thereby triggering the release of inflammatory cytokines (such as TNF-α and IL-6). This suppresses triglyceride hydrolysis in chylomicrons and LDL while downregulating Apo-A1 expression, impairing reverse cholesterol transport (11, 38); (2) HSC activation: copper-dependent oxidative stress synergizes with lipotoxicity to activate HSCs, thereby promoting TGF-β1-mediated excessive deposition in the extracellular matrix (39–41); (3) endoplasmic reticulum stress and mitophagy dysfunction: these mechanisms amplify the vicious cycle of “metabolic dysregulation-inflammation-fibrosis” (42). Thus, dynamic monitoring of HDL-C and TG levels not only provides early risk stratification for hepatic fibrosis in WD patients but also suggests potential therapeutic targets for combined copper chelation (such as D-penicillamine) and lipid metabolism modulation.

Gender disparity was identified as another critical predictive factor. Specifically, it modulates the pathological progression of “copper-lipid co-toxicity” through sex hormone-inflammatory signaling interplay. This study demonstrates that male WD patients, lacking estrogen-mediated protection, are more susceptible to HDL-C/TG metabolic dysregulation and accelerated fibrosis progression. Clinical studies support these findings, demonstrating significantly higher incidences of end-stage hepatic fibrosis, cirrhosis, and HCC in males compared to females (43). This sex-based divergence may stem from estrogen-regulated antifibrotic pathways: estrogen suppresses IL-6 autocrine signaling via estrogen receptor alpha (ERα) activation, thereby blocking the IL-6/signal transducer and activator of transcription 3 (STAT3) inflammatory axis (44). Mechanistically, IL-6 binds to its receptor (IL-6R) and activates the JAK kinases, inducing STAT3 phosphorylation (45). Phosphorylated STAT3 forms dimers that translocate to the nucleus, driving profibrogenic gene expression (such as TGF-β1 and Collagen I) and acute-phase protein synthesis (46). In WD, copper accumulation enhances activation of the IL-6/STAT3 signaling pathway, promoting inflammatory and fibrogenic responses. Concurrently, the ERα complex exerts a counter-regulatory effect by competitively inhibiting IL-6 promoter activity, thereby suppressing Kupffer cell–derived IL-6 secretion and mitigating pathological STAT3 signaling (47, 48), establishing a “metabolic-sex hormone” counteractive equilibrium. Consequently, estrogen’s suppression of IL-6/STAT3 in females mitigates copper-lipid co-toxicity-driven inflammatory amplification. Conversely, in males—devoid of this protective mechanism—exhibit accelerated transition from metabolic dysregulation to ECM deposition. This interaction network provides a rationale for sex-specific therapies, such as STAT3 inhibitors or selective estrogen receptor modulators.

Despite the promising and valuable findings, this study has several limitations that must be acknowledged. First, the absence of WD-specific reference thresholds for advanced hepatic fibrosis based on FibroScan necessitated the adoption of thresholds from the MAFLD, which shares overlapping pathological mechanisms. However, these thresholds lack standardized criteria and histological validation via liver biopsy, potentially influencing observational accuracy. Second, despite comprehensive data collection, incomplete records from some patients due to early-stage initial visits may have introduced bias. And then, although we performed a bootstrap validation on the entire dataset in order to strengthen the validation, there are overfitting and optimism bias as result of multiple variables, data noise and stochastic variations in our predictive models. Finally, the single center design and absence of external validation limit the generalizability of the finding, necessitating confirmation through multicenter, large-scale studies to ensure broader clinical applicability.

## 5 Conclusion

In conclusion, CER, Apo-A1, LN, PLT, HDL-C, TG, and Sex were identified as independent risk factors for advanced hepatic fibrosis in WD patients undergoing long-term maintenance therapy. The predictive model established based on these factors demonstrated exceptional discriminative ability, high calibration accuracy, clinical utility, and biological plausibility (rationality analysis). These findings provide clinicians with a quantitative and intuitive tool for assessing the risk of advanced hepatic fibrosis.

## Data Availability

The raw data supporting the conclusions of this article will be made available by the authors, without undue reservation.

## References

[1] Mulligan C Bronstein J. Wilson disease: an overview and approach to management. *Neurol Clin.* (2020) 38:417–32. 10.1016/j.ncl.2020.01.005 32279718

[2] Himoto T Masaki T. Current trends of essential trace elements in patients with chronic liver diseases. *Nutrients.* (2020) 12:2084. 10.3390/nu12072084 32674425 PMC7400835

[3] Roberts E. Update on the diagnosis and management of wilson disease. *Curr Gastroenterol Rep.* (2018) 20:56. 10.1007/s11894-018-0660-7 30397835

[4] Gerosa C Fanni D Congiu T Piras M Cau F Moi M Liver pathology in Wilson’s disease: from copper overload to cirrhosis. *J Inorg Biochem.* (2019) 193:106–11. 10.1016/j.jinorgbio.2019.01.008 30703747

[5] Žigrai M Vyskočil M Tóthová A Vereš P Bluska P Valkovič P. Late-onset Wilson’s disease. *Front Med (Lausanne).* (2020) 7:26. 10.3389/fmed.2020.00026 32118011 PMC7016193

[6] Trautwein C Friedman S Schuppan D Pinzani M. Hepatic fibrosis: concept to treatment. *J Hepatol.* (2015) 62:S15–24. 10.1016/j.jhep.2015.02.039 25920084

[7] Zhang C Yuan W He P Lei J Wang C. Liver fibrosis and hepatic stellate cells: etiology, pathological hallmarks and therapeutic targets. *World J Gastroenterol.* (2016) 22:10512–22. 10.3748/wjg.v22.i48.10512 28082803 PMC5192262

[8] Jinqiu Y Wenxia Z Cheng Z Tong L. Risk factors for the development of advanced liver fibrosis in nonalcoholic fatty liver disease and establishment of a nomogram model. *J Clin Hepatol.* (2024) 40:1579–84. 10.12449/JCH240812

[9] European Association for the Study of the Liver. Easl clinical practice guidelines on non-invasive tests for evaluation of liver disease severity and prognosis - 2021 update. *J Hepatol.* (2021) 75:659–89. 10.1016/j.jhep.2021.05.025 34166721

[10] Abedi V Avula V Chaudhary D Shahjouei S Khan A Griessenauer C Prediction of long-term stroke recurrence using machine learning models. *J Clin Med.* (2021) 10:1286. 10.3390/jcm10061286 33804724 PMC8003970

[11] Zhao C Dong T Sun L Hu H Wang Q Tian L [Establishment and validation of a predictive nomogram for liver fibrosis in patients with wilson disease and abnormal lipid metabolism]. *Nan Fang Yi Ke Da Xue Xue Bao.* (2022) 42:1720–5. 10.12122/j.issn.1673-4254.2022.11.17 36504066 PMC9742779

[12] Tao Z Yang P Zhou J Wang R Jiang Z Han H Ideal serum non-ceruloplasmin bound copper prediction for long-term treated patients with wilson disease: a nomogram model. *Front Med (Lausanne).* (2023) 10:1275242. 10.3389/fmed.2023.1275242 38020085 PMC10656596

[13] Chinese Society of Hepatology, Chinese Medical Association. [Guidelines for the prevention and treatment of metabolic dysfunction-associated (non-alcoholic) fatty liver disease (version 2024)]. *Zhonghua Gan Zang Bing Za Zhi.* (2024) 32:418–34. 10.3760/cma.j.cn501113-20240327-00163 38858192 PMC12677420

[14] Inherited Metabolic Liver Disease Collaboration Group, Chinese Society of Hepatology, Chinese Medical Association. [Guidelines for the diagnosis and treatment of hepatolenticular degeneration (2022 edition)]. *Zhonghua Gan Zang Bing Za Zhi.* (2022) 30:9–20. 10.3760/cma.j.cn501113-20211217-00603 35152665 PMC12770193

[15] Liggi M Murgia D Civolani A Demelia E Sorbello O Demelia L. The Relationship between copper and steatosis in wilson’s disease. *Clin Res Hepatol Gastroenterol.* (2013) 37:36–40. 10.1016/j.clinre.2012.03.038 22572525

[16] Day C James O. Steatohepatitis: a tale of two “hits”? *Gastroenterology.* (1998) 114:842–5. 10.1016/s0016-5085(98)70599-2 9547102

[17] Molenaar M Vaandrager A Helms J. Some lipid droplets are more equal than others: different metabolic lipid droplet pools in hepatic stellate cells. *Lipid Insights.* (2017) 10:1178635317747281. 10.1177/1178635317747281 29276391 PMC5734559

[18] Eslam M Sarin S Wong V Fan J Kawaguchi T Ahn S The Asian pacific association for the study of the liver clinical practice guidelines for the diagnosis and management of metabolic associated fatty liver disease. *Hepatol Int.* (2020) 14:889–919. 10.1007/s12072-020-10094-2 33006093

[19] Zeng D Liu Y Zhang J Zhu Y Lin S You J Serum ceruloplasmin levels correlate negatively with liver fibrosis in males with chronic hepatitis b: a new noninvasive model for predicting liver fibrosis in hbv-related liver disease. *PLoS One.* (2013) 8:e0077942. 10.1371/journal.pone.0077942 24282481 PMC3837017

[20] Yin H Lin Z Nie S Wu J Tan Z Zhu J Mass-selected site-specific core-fucosylation of ceruloplasmin in alcohol-related hepatocellular carcinoma. *J Proteome Res.* (2014) 13:2887–96. 10.1021/pr500043k 24799124 PMC4059274

[21] Poynard T Abella A Pignon J Naveau S Leluc R Chaput J. Apolipoprotein AI and alcoholic liver disease. *Hepatology.* (1986) 6:1391–5. 10.1002/hep.1840060628 3098667

[22] Deckmyn O Poynard T Bedossa P Paradis V Peta V Pais R Clinical interest of serum Alpha-2 macroglobulin, apolipoprotein A1, and haptoglobin in patients with non-alcoholic fatty liver disease, with and without type 2 diabetes, before or during covid-19. *Biomedicines.* (2022) 10:699. 10.3390/biomedicines10030699 35327501 PMC8945355

[23] Poynard T Bedossa P Mathurin P Ratziu V Paradis V. Apolipoprotein A1 and hepatic fibrosis. *J Hepatol.* (1995) 22:107–10. 7665844

[24] Chen Q Mei L Zhong R Han P Wen J Han X Serum liver fibrosis markers predict hepatic decompensation in compensated cirrhosis. *BMC Gastroenterol.* (2023) 23:317. 10.1186/s12876-023-02877-2 37726681 PMC10510279

[25] Aldinger K Mosca S Tétreault M Dempsey J Ishak G Hartley T Mutations in Lama1 cause cerebellar dysplasia and cysts with and without retinal dystrophy. *Am J Hum Genet.* (2014) 95:227–34. 10.1016/j.ajhg.2014.07.007 25105227 PMC4129402

[26] Fu Y Zhou Y Mu Y Lv Y Chen G Zhang H Testicular orphan receptor 4 induced hepatic stellate cells activation via the regulation of Tgf-B receptor ?/Smad2/3 signaling pathway. *Ann Hepatol.* (2023) 28:100775. 10.1016/j.aohep.2022.100775 36280014

[27] Menu Y Vilgrain V Asselain B Scherrer A Sellier N Thonnart B [Value of a score using clinical and biological variables for determining the benignity or malignancy of a hepatic mass]. *Gastroenterol Clin Biol.* (1989) 13:340–2. 2661292

[28] Xiaomin L Huiying S Yongfu W Fuai L. The significance of ha, col?, ln and P?np-P in the evaluation of connective tissue disease with interstitial lung disease. *Tianjin Med J.* (2021) 49:617–21. 10.11958/20210036

[29] Moore A. Thrombocytopenia in cirrhosis: a review of pathophysiology and management options. *Clin Liver Dis (Hoboken).* (2019) 14:183–6. 10.1002/cld.860 31879561 PMC6924969

[30] Gallo P Terracciani F Di Pasquale G Esposito M Picardi A Vespasiani-Gentilucci U. Thrombocytopenia in chronic liver disease: physiopathology and new therapeutic strategies before invasive procedures. *World J Gastroenterol.* (2022) 28:4061–74. 10.3748/wjg.v28.i30.4061 36157107 PMC9403422

[31] Lesurtel M Graf R Aleil B Walther D Tian Y Jochum W Platelet-derived serotonin mediates liver regeneration. *Science.* (2006) 312:104–7. 10.1126/science.1123842 16601191

[32] Zaldivar M Pauels K von Hundelshausen P Berres M Schmitz P Bornemann J Cxc chemokine ligand 4 (Cxcl4) is a platelet-derived mediator of experimental liver fibrosis. *Hepatology.* (2010) 51:1345–53. 10.1002/hep.23435 20162727

[33] Aryal B Yamakuchi M Shimizu T Kadono J Furoi A Gejima K Therapeutic implication of platelets in liver regeneration -hopes and hues. *Expert Rev Gastroenterol Hepatol.* (2018) 12:1219–28. 10.1080/17474124.2018.1533813 30791793

[34] Takahashi K Liang C Oda T Ohkohchi N. Platelet and liver regeneration after liver surgery. *Surg Today.* (2020) 50:974–83. 10.1007/s00595-019-01890-x 31720801

[35] Matsuo R Ohkohchi N Murata S Ikeda O Nakano Y Watanabe M Platelets strongly induce hepatocyte proliferation with Igf-1 and Hgf in vitro. *J Surg Res.* (2008) 145:279–86. 10.1016/j.jss.2007.02.035 17688880

[36] Santoleri D Titchenell P. Resolving the paradox of hepatic insulin resistance. *Cell Mol Gastroenterol Hepatol.* (2019) 7:447–56. 10.1016/j.jcmgh.2018.10.016 30739869 PMC6369222

[37] Lucero D Miksztowicz V Macri V López G Friedman S Berg G Overproduction of altered Vldl in an Insulin-resistance rat model: influence of Srebp-1c and Ppar-A. *Clin Investig Arterioscler.* (2015) 27:167–74. 10.1016/j.arteri.2014.11.002 25796423

[38] Xin L Wei Y Yu Z. Clinical significance of Apob/Apoa1 and 25-(Oh)D in predicting the severity of primary biliary cirrhosis. *China J Modern Med.* (2023) 33:66–71. 10.3969/j.issn.1005-8982.2023.02.011

[39] Wu P Dong J Cheng N Yang R Han Y Han Y. Inflammatory cytokines expression in Wilson’s disease. *Neurol Sci.* (2019) 40:1059–66. 10.1007/s10072-018-3680-z 30644005

[40] Gandhi C. Oxidative stress and hepatic stellate cells: a paradoxical relationship. *Trends Cell Mol Biol.* (2012) 7:1–10. 27721591 PMC5051570

[41] Carotti S Aquilano K Valentini F Ruggiero S Alletto F Morini S An overview of deregulated lipid metabolism in nonalcoholic fatty liver disease with special focus on lysosomal acid lipase. *Am J Physiol Gastrointest Liver Physiol.* (2020) 319:G469–80. 10.1152/ajpgi.00049.2020 32812776

[42] Higashi T Friedman S Hoshida Y. Hepatic stellate cells as key target in liver fibrosis. *Adv Drug Deliv Rev.* (2017) 121:27–42. 10.1016/j.addr.2017.05.007 28506744 PMC5682243

[43] He Y Guo X Lan T Xia J Wang J Li B Correction to: human umbilical cord-derived mesenchymal stem cells improve the function of liver in rats with acute-on-chronic liver failure via downregulating notch and stat1/stat3 signaling. *Stem Cell Res Ther.* (2022) 13:65. 10.1186/s13287-022-02728-z 35130963 PMC8822683

[44] Stärkel P Schnabl B Leclercq S Komuta M Bataller R Argemi J Deficient Il-6/Stat3 signaling, high Tlr7, and type I interferons in early human alcoholic liver disease: a triad for liver damage and fibrosis. *Hepatol Commun.* (2019) 3:867–82. 10.1002/hep4.1364 31334440 PMC6601428

[45] Li H Liu N Li J Wang M Tan J Dong B Bicyclol ameliorates advanced liver diseases in murine models via inhibiting the Il-6/Stat3 signaling pathway. *Biomed Pharmacother.* (2022) 150:113083. 10.1016/j.biopha.2022.113083 35658240

[46] Hongmei H Sanqiang L Huajie L Yongyong Z Xiuxiu W Zhao M. Effect of Il-6 trans-signialing on expression of Pcna during acute liver injury induced by acetaminophen in mice. *Chinese J Gastroenterol Hepatol.* (2016) 25:1005–8. 10.1002/jbt.21708 25914167

[47] Palmisano B Zhu L Stafford J. Role of estrogens in the regulation of liver lipid metabolism. *Adv Exp Med Biol.* (2017) 1043:227–56. 10.1007/978-3-319-70178-3_12 29224098 PMC5763482

[48] Meda C Barone M Mitro N Lolli F Pedretti S Caruso D Hepatic Erα accounts for sex differences in the ability to cope with an excess of dietary lipids. *Mol Metab.* (2020) 32:97–108. 10.1016/j.molmet.2019.12.009 32029233 PMC6957843

