# Supplementary material for: Risk factor analysis and nomogram development for advanced-stage hepatic fibrosis in patients with Wilson’s disease
Source: Front Med (Lausanne). 2025 Jul 30;12:1650584. doi: 10.3389/fmed.2025.1650584 (PMC12343619; doi:10.3389/fmed.2025.1650584)
Supplement: Supplementary file 1 [file Supplementary_file_1.docx]

Supplementary Material

# Supplementary Figures

**
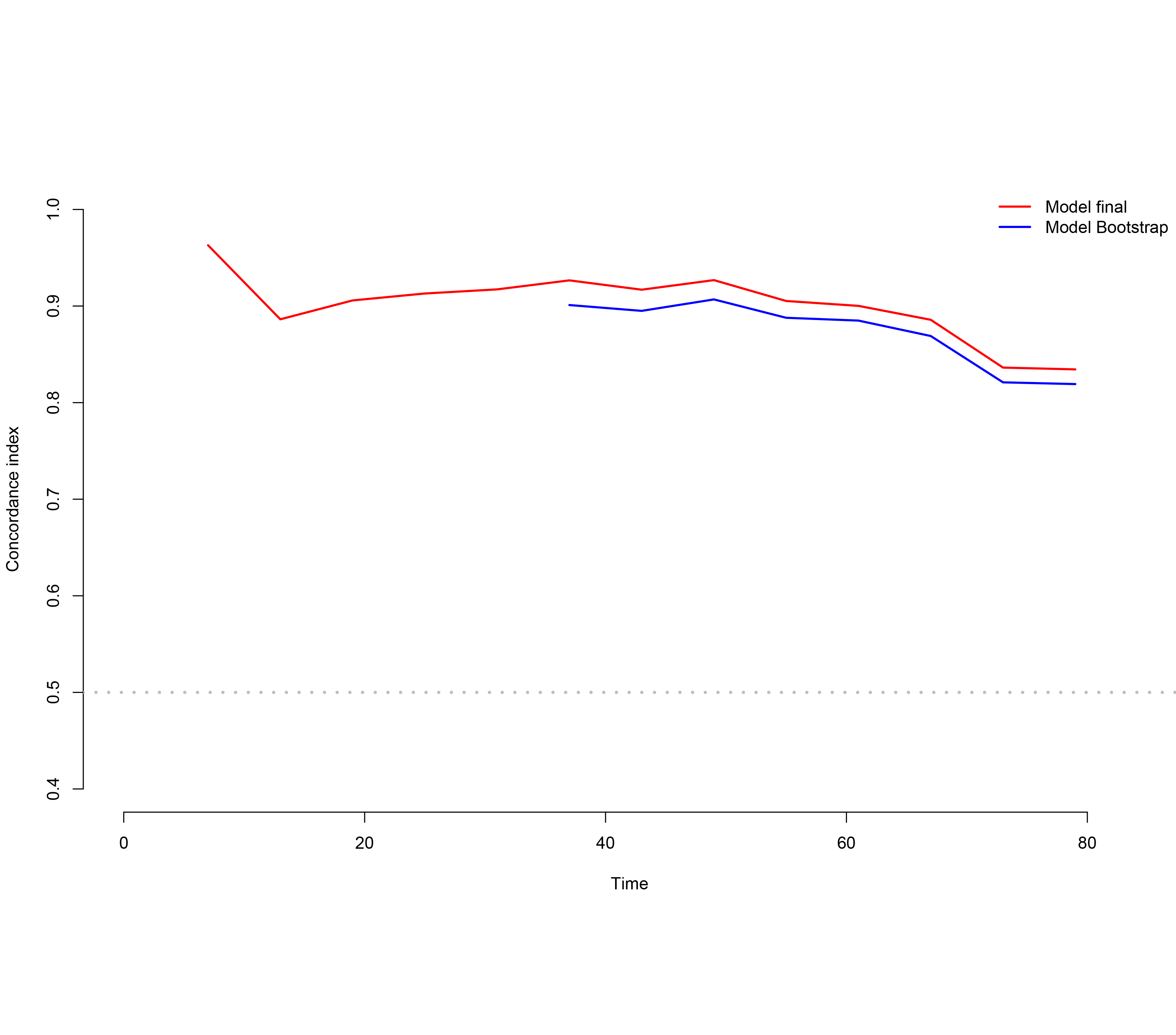
Supplementary Figure 1.** Bootstrap validation on the entire dataset. The C-index remains stable between 0.80 and 0.90 with 95% confidence intervals (0.812-0.902).
